# POLARIS: A phase 2 trial of encorafenib plus binimetinib evaluating high-dose and standard-dose regimens in patients with *BRAF* V600-mutant melanoma with brain metastasis

**DOI:** 10.1093/noajnl/vdae033

**Published:** 2024-03-18

**Authors:** Alexander M Menzies, Georgina V Long, Amiee Kohn, Hussein Tawbi, Jeffrey Weber, Keith Flaherty, Grant A McArthur, Paolo A Ascierto, Yanina Pfluger, Karl Lewis, Katy K Tsai, Omid Hamid, Hans Prenen, Luis Fein, Erjian Wang, Carolin Guenzel, Fan Zhang, Joseph F Kleha, Alessandra di Pietro, Michael A Davies

**Affiliations:** Melanoma Institute Australia, NSW, Australia, and The University of Sydney, Sydney, Australia; Royal North Shore and Mater Hospitals, The University of Sydney, Sydney, Australia; Melanoma Institute Australia, NSW, Australia, and The University of Sydney, Sydney, Australia; Royal North Shore and Mater Hospitals, The University of Sydney, Sydney, Australia; Division of Hematology/Medical Oncology, School of Medicine, Oregon Health Sciences University, Portland, Oregon, USA; Department of Melanoma Medical Oncology, University of Texas MD Anderson Cancer Center, Houston, Texas, USA; Laura and Isaac Perlmutter Cancer Center, NYU Langone Health, New York, New York, USA; Massachusetts General Cancer Center, Massachusetts General Hospital, Boston, Massachusetts, USA; Sir Peter MacCallum Department of Oncology, Peter MacCallum Cancer Centre, University of Melbourne, Melbourne, Victoria, Australia; Unit of Melanoma Cancer Immunotherapy and Development Therapeutics, Istituto Nazionale Tumori IRCCS Fondazione Pascale, Napoli, Italy; Alexander Fleming Institute, Buenos Aires, Argentina; Medical Oncology, University of Colorado, Health Center, Denver, Colorado, USA; Helen Diller Family Comprehensive Cancer Center, University of California San Francisco, San Francisco, California, USA; The Angeles Clinic and Research Institute, A Cedars-Sinai Affiliate Los Angeles, California, USA; Oncology Department, University Hospital Antwerp, Antwerp, Belgium; Alexander Fleming Institute, Buenos Aires, Argentina; Formerly Pfizer, New York, New York, USA; Formerly Pfizer, New York, New York, USA; Formerly Pfizer, New York, New York, USA; Formerly Pfizer, New York, New York, USA; Pfizer Srl, Milan, Italy; Department of Melanoma Medical Oncology, University of Texas MD Anderson Cancer Center, Houston, Texas, USA

**Keywords:** Binimetinib, *BRAF* V600, Encorafenib, Melanoma, POLARIS

## Abstract

**Background:**

POLARIS (phase 2 [ph2]; NCT03911869) evaluated encorafenib (BRAF inhibitor) in combination with binimetinib (MEK1/2 inhibitor) in BRAF/MEK inhibitor-naïve patients with *BRAF* V600-mutant melanoma with asymptomatic brain metastases.

**Methods:**

The safety lead-in (SLI) assessed tolerability for high-dose encorafenib 300 mg twice daily (BID) plus binimetinib 45 mg BID. If the high dose was tolerable in ph2, patients would be randomized to receive high or standard dose (encorafenib 450 mg once daily [QD] plus binimetinib 45 mg BID). Otherwise, standard dose was evaluated as the recommended ph2 dose (RP2D). Patients who tolerated standard dosing during Cycle 1 could be dose escalated to encorafenib 600 mg QD plus binimetinib 45 mg BID in Cycle 2. Safety, efficacy, and pharmacokinetics were examined.

**Results:**

RP2D was standard encorafenib dosing, as >33% of evaluable SLI patients (3/9) had dose-limiting toxicities. Overall, of 13 safety-evaluable patients (10 SLI, 3 ph2), 9 had prior immunotherapy. There were 9 treatment-related adverse events in the SLI and 3 in ph2. Of the SLI efficacy-evaluable patients (*n* = 10), 1 achieved complete response and 5 achieved partial responses (PR); the brain metastasis response rate (BMRR) was 60% (95% CI: 26.2, 87.8). In ph2, 2 of 3 patients achieved PR (BMRR, 67% [95% CI: 9.4, 99.2]). Repeated encorafenib 300 mg BID dosing did not increase steady-state exposure compared with historical 450 mg QD data.

**Conclusions:**

Despite small patient numbers due to early trial termination, BMRR appeared similar between the SLI and ph2, and the ph2 safety profile appeared consistent with previous reports of standard-dose encorafenib in combination with binimetinib.

## Lay Summary

Melanoma is an aggressive form of skin cancer that often spreads to the brain. Patients have a poor prognosis, especially for those who failed to respond to immunotherapy. Previous trials of targeted drug therapies that block an overactive growth pathway in melanoma cells containing a BRAF V600-mutation have demonstrated responses but with short durability. Higher targeted therapy doses might overcome resistance and improve duration of benefit. The POLARIS clinical trial tested the approved combination of encorafenib plus binimetinib, initially testing a higher dose of encorafenib than is standard. Treatment with the higher dose was toxic despite not increasing drug exposure, possibly related to an interaction with prior immunotherapy. Clinical response rate was similar to previous regimens, however only 13 patients were enrolled.

**Key Points**

- POLARIS evaluated high-/standard-dose encorafenib in combination with binimetinib.
- Encorafenib in combination with binimetinib showed intracranial activity.
- Brain metastasis response rate was similar in the high-dose safety lead-in and phase 2 with standard dosing.

**Importance of the Study**

POLARIS is the first study to explore encorafenib combined with binimetinib that aimed to assess a high-dose regimen for patients with *BRAF* V600-mutant melanoma with brain metastasis. Administration of a higher encorafenib dose (300 mg, twice daily) did not yield increased drug exposure, potentially due to heightened drug metabolism. Despite the lack of increased drug exposure, high-dose encorafenib combined with binimetinib (45 mg, twice daily) was not well tolerated, while tolerability of the standard encorafenib dose (450 mg, once daily) combined with binimetinib was in line with prior investigations, with similar pharmacokinetics and no new safety signals. These 3 encorafenib dosage levels exhibited signs of intracranial activity; however, the duration of response was limited. The small number of patients in the study limits interpretation. The POLARIS study supports the remaining unmet need for patients with melanoma with brain metastases, particularly following the progression of immunotherapy.

In unresectable or metastatic melanoma with a *BRAF* V600-activating mutation, combination regimens targeting BRAF and MEK1/MEK2 have shown long-term clinical benefit and are recommended therapy options.^1–9^ Approximately 50% of metastatic melanomas have point mutations in *BRAF*, with the most common, V600E, present in about 85% of *BRAF*-mutated melanomas.^10^ *BRAF* V600 mutations activate the mitogen-activated protein kinase (MAPK) pathway downstream of RAS, leading to tumor proliferation. Upon treatment with BRAF inhibitor monotherapy, patients with *BRAF* V600-mutant tumors frequently develop resistance via MAPK pathway reactivation.^11–16^ Combining BRAF and MEK1/MEK2 inhibitors delays resistance and reduces the development of secondary malignancies compared with BRAF inhibitors alone.^17^

Approximately 43%–75% of patients with advanced melanoma develop brain metastases.^18,19^ Although melanoma brain metastases have been historically associated with poor survival outcomes, this has markedly improved in recent years with stereotactic radiosurgery and combination checkpoint inhibitor therapy, resulting in increased 1-year overall survival rates from approximately 25% to 85%.^20–23^ Even though checkpoint inhibitor therapy has demonstrated intracranial activity with durable benefits, toxicity rates can be a concern,^22–24^ suggesting a need for alternative or combination treatments.

Clinical trials of BRAF/MEK inhibition for brain metastases have shown clinical utility.^25^ The COMBI-MB trial (NCT02039947) demonstrated that dabrafenib (BRAF inhibitor) plus trametinib (MEK1/2 inhibitor) has activity in patients with *BRAF* V600-mutant melanoma brain metastases and a manageable safety profile. While extracranial responses were consistent with those of patients without brain metastases, the intracranial duration of response (DOR) was shorter (patients with a *BRAF* V600E mutation and melanoma brain metastases, without prior local brain-directed therapy [median DOR, 6.5 months; 95% CI: 4.9, 10.3]) than extracranial response duration (median DOR, 10.2 months; 95% CI: 5.8, not estimable).^26^

Encorafenib is a small-molecule ATP-competitive BRAF inhibitor.^27^ In the phase 3 COLUMBUS trial (NCT01909453), co-administration of standard-dose encorafenib 450 mg once daily (QD) with the MEK1/2 inhibitor binimetinib 45 mg twice daily (BID) demonstrated efficacy in patients with *BRAF* V600-mutant melanoma with generally reversible and manageable adverse events (AEs) and a relatively low rate of treatment discontinuation due to AEs (16%–18%^3,5^; the most common were alanine aminotransferase, aspartate aminotransferase, blood creatinine, headache, and rash).^3,5^

Encorafenib is a P-glycoprotein (P-gpl, an efflux transporter on the blood–brain barrier) substrate and an inhibitor of breast cancer resistance protein (BCRP, an efflux transporter on the blood–brain barrier), and binimetinib is a P-gpl and BCRP substrate.^28–31^ Although both have high-intrinsic membrane permeability, efflux transporters may result in reduced concentrations of the drugs in the brain. The role of a potentially compromised blood–brain barrier in treating patients with brain metastases is not well understood, and thus evaluating a high-dose regimen in these patients may help overcome potential limitations in brain penetration compared with the standard-dose regimen. We hypothesized that treatment with encorafenib at a higher dose (300 mg BID), compared with the standard dose of 450 mg QD, may increase the daily area under the curve and trough concentration (C_trough_) by 33% and 700%, respectively, resulting in increased exposure and, in turn, higher brain exposure. The phase 1 study evaluated a dose of 600 mg QD; however, this dose was not selected as the maximum tolerated dose due to rare renal toxicity.^32^ We, therefore, assessed whether a dose of 300 mg BID, rather than 600 mg QD, could reduce some toxicities that have been associated with peak concentrations (eg, visual changes).^32^

Here, we report on the phase 2 POLARIS trial (NCT03911869), which prospectively evaluated encorafenib in combination with binimetinib in BRAF/MEK inhibitor-naive patients with *BRAF* V600-mutant melanoma with asymptomatic brain metastases, including those who had progressed on prior checkpoint immunotherapy. In addition to the standard dose, high-dose encorafenib was explored as its efficacy and safety remain to be determined.

## Methods

### Experimental Model and Patient Details

#### Ethical statement.—

The final protocol, amendments, and informed consent documentation were reviewed and approved by institutional review boards and independent ethics committees at each participating center. Investigators were required to inform their institutional review boards or independent ethics committees of the study’s progress and the occurrence of any serious or unexpected AEs. This study was conducted in compliance with the Declaration of Helsinki, the International Conference on Harmonization Guideline for Good Clinical Practice, and applicable local regulatory requirements.

### Methods

#### Study design and patients.—

**Figure 1.**
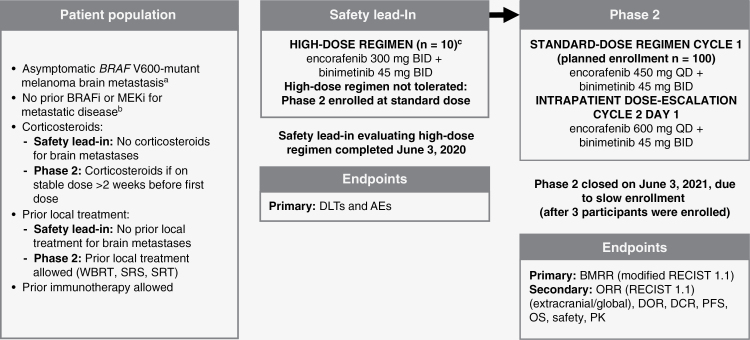
POLARIS study design flow chart. A randomized, multicenter, open-label, phase 2 study evaluating a safety lead-in high-dose (encorafenib 300 mg BID + binimetinib 45 mg BID) and phase 2 standard-dose (encorafenib 450 mg QD + binimetinib 45 mg BID) with intrapatient dose escalation (encorafenib 600 mg QD + binimetinib 45 mg BID) in patients with *BRAF* V600-mutant melanoma with brain metastases. AE, adverse event; BID, twice daily; BMRR, brain metastasis response rate; BRAFi, BRAF inhibitor; DCR, duration of complete response; DLT, dose-limiting toxicity; DOR, duration of response; MEKi, MEK inhibitor; OS, overall survival; PFS, progression-free survival; PK, pharmacokinetics; QD, once daily; RECIST, Response Evaluation Criteria in Solid Tumors; SRS, stereotactic radiosurgery; SRT, superficial radiation therapy; WBRT, whole-brain radiotherapy. ^a^At least 1 brain lesion ≥0.5 cm and ≤4 cm. ^b^BRAFi/MEKi, safety lead-in ≥12 months adjuvant setting allowed; phase 2 ≥6 months adjuvant setting allowed. ^c^Evaluable patients must have a DLT or have received ≥75% of the planned cumulative dose of both study drugs during Cycle 1 (28 days).

POLARIS is an open-label, multicenter phase 2 trial that included a safety lead-in (SLI) to determine the recommended phase 2 dose (RP2D), and assessment of safety, efficacy, and pharmacokinetics for 2 dosing regimens of encorafenib in combination with binimetinib in patients with *BRAF* V600-mutant melanoma with asymptomatic brain metastases (Figure 1). In the SLI, tolerability of high-dose encorafenib 300 mg BID in combination with binimetinib 45 mg BID was determined based on the rate of dose-limiting toxicities (DLTs) (Figure 1). If the high-dose regimen was determined to be safe based on the SLI results, the stratified randomized study design provided an unbiased approach to evaluate the efficacy and safety of 2 dosing regimens. If the high-dose regimen was determined not to be safe based on the SLI results, then the standard dose would be considered the RP2D in phase 2. If the RP2D was the standard dose, patients able to tolerate the standard dose during the first 4 weeks of phase 2 treatment (Cycle 1) were escalated to encorafenib 600 mg QD in combination with binimetinib 45 mg BID (Figure 1; additional details in Supplementary Data).

Key eligibility criteria included written informed consent, age ≥18 years, ECOG PS 0 or 1, Karnofsky score ≥80, no prior BRAF or MEK inhibitor treatment for unresectable metastatic disease, histologically confirmed diagnosis of cutaneous melanoma with metastases to the brain, measurable intracranial disease by modified RECIST 1.1, presence of *BRAF* V600 mutation in tumor tissue previously determined by a local polymerase chain reaction or next-generation sequencing-based assay before screening or a central laboratory during screening. Key exclusion criteria included symptomatic brain metastasis (ie, neurological symptoms), uveal or mucosal melanoma, and history of or current leptomeningeal metastases. Additional details on key inclusion and exclusion criteria are provided in Supplementary Data.

#### Safety analysis.—

Safety data were summarized descriptively (see additional details in Supplementary Data).

#### Pharmacokinetic analysis.—

Pharmacokinetic (PK) data were summarized descriptively (see additional details in Supplementary Data).

#### Statistical analysis.—

If the sponsor and investigators determined that the SLI high-dose treatment was safe in the first 9 evaluable (completed Cycle 1 [28 days] of treatment and received at least 75% of the planned cumulative dose of both study drugs or experienced a DLT) patients enrolled, randomization would start for the phase 2 portion of the study. Phase 2 was designed to test the null hypothesis of brain metastasis response rate (BMRR) of ≤30%, which is considered not clinically meaningful and insufficient to warrant further study, against the alternative hypothesis of true BMRR of ≥50%. If the high-dose regimen was tolerated in the SLI, ≈50 patients each in the high- and standard-dose arms were planned to be randomized for phase 2 to provide approximately 90% power at a 1-sided 5% significance level; if not, ≤100 patients were planned to be enrolled in the standard-dose arm.

For the SLI, the number and proportion of patients in the high-dose SLI experiencing DLTs during the first treatment cycle were summarized descriptively.

The primary endpoint of the phase 2 study was the BMRR, which was defined as the proportion of total patients who had achieved the best overall brain metastasis confirmed complete response (CR) or partial response (PR) according to the modified Response Evaluation Criteria in Solid Tumors, version 1.1 per investigator assessment^33,34^ (see additional details in Supplementary Data).

## Results

### Baseline Characteristics

The study enrolled 13 patients, including 10 in the SLI and 3 in phase 2 (phase 2 was terminated early due to slow enrollment). The median age of patients in the SLI and phase 2 parts was 66.5 years (range, 39–83) and 48.0 years (range, 39–50), respectively (Table 1). The median time from initial cancer diagnosis to study entry for patients in the SLI and phase 2 parts was 18.3 months (range, 1.3–22.3) and 78.2 months (range, 49.0–87.6), respectively. In the SLI, 6 patients (60%) had 1 or 2 brain lesions (0.5–≤4 cm); 2 of 3 patients in phase 2 had ≥3 brain lesions. The median lactase dehydrogenase level for patients in the SLI was 3.3 times the upper limit of normal (range, 2.5–5.3) and 7.7 times the upper limit of normal (range, 3.5–56.8) in phase 2 (Table 1).

**Table 1.** Baseline characteristics

	All Patients, *N* = 13	Safety Lead-In, *n* = 10	Phase 2, *n* = 3
Age, median, y (range)	56.0 (39.0–83.0)	66.5 (39.0–83.0)	48.0 (39.0–50.0)
<65 y, *n* (%)	8 (61.5)	5 (50.0)	3 (100)
Sex, *n* (%)			
Male	10 (76.9)	8 (80.0)	2 (66.7)
Race^a^, *n* (%)			
White	12 (92.3)	9 (90.0)	3 (100)
Not reported due to confidentiality regulations	1 (7.7)	1 (10.0)	0
ECOG PS, *n* (%)^b^			
0	8 (61.5)	7 (70.0)	1 (33.3)
1	5 (38.5)	3 (30.0)	2 (66.7)
*BRAF* genotype, *n* (%)			
* V600E*	9 (69.2)	6 (60.0)	3 (100)
* V600K*	3 (23.1)	3 (30.0)	0
No mutation detected	1 (7.7)	1 (10.0)	0
Time since initial cancer diagnosis, median, months^c^ (range)	26.2 (1.3–222.3)	18.3 (1.3–222.3)	78.2 (49.0–87.6)
No. of brain metastases, *n* (%)			
1–2 lesions	7 (53.8)	6 (60.0)	1 (33.3)
≥3 lesions	6 (46.2)	4 (40.0)	2 (66.7)
LDH level (×ULN) at baseline, median (range)	3.5 (2.5–56.8)	3.3 (2.5–5.3)	7.7 (3.5–56.8)
Received prior radiotherapy for brain metastases	1 (7.7)	0	1 (33.3)
Prior systemic therapy, *n* (%)			
0	3 (23.1)	1 (10.0)	2 (66.7)
1	9 (69.2)	8 (80.0)	1 (33.3)
2	1 (7.7)	1 (10.0)	0
Prior anticancer therapy, *n* (%)^d^			
Any	10 (76.9)	9 (90.0)	1 (33.3)
Nivolumab	8 (61.5)	8 (80.0)	0
Ipilimumab + nivolumab	1 (7.7)	1 (10.0)	0
Other^e^	3 (23.1)	2 (20.0)	1 (33.3)
Duration of prior anticancer therapy, median (range), months			
Any	7.0	4.4	12.5
Nivolumab	7.0	7.0	NA
Ipilimumab	0.7	0.7	NA
Time from end of last anticancer therapy to start of study, median months			
Any	1.8	1.2	23.3
Nivolumab	1.8	1.8	NA
Ipilimumab	1.2	1.2	NA
Patients with concomitant steroid medications, *n* (%)^d^^,^^f^			
Any	7 (53.8)	6 (60.0)	1 (33.3)
Prednisone	5 (38.5)	5 (50.0)	0
Methylprednisolone	2 (15.4)	1 (10.0)	1 (33.3)
Dexamethasone	1 (7.7)	1 (10.0)	0
Prednisolone	1 (7.7)	0	1 (33.3)

ECOG PS, Eastern Cooperative Oncology Group performance status; LDH, lactate dehydrogenase; NA, not applicable; SLD, sum of longest diameters; ×ULN, times the upper limit of normal.

^a^Because >1 race can be selected, a patient can be counted in multiple rows.

^b^0 = without restriction; 1 = restricted in physically strenuous activity but ambulatory and able to carry out work of a light or sedentary nature (eg, light housework, office work).

^c^Time since initial diagnosis is defined as (date of first dose - date of initial diagnosis)/30.4375.

^d^Percentages do not add up to 100% because some patients received >1 treatment.

^e^Other treatments were investigational drugs and talimogene laherparepvec.

^f^Steroid use was recorded as concomitant medication, but the reason for its use was not collected.

None of the enrolled patients had prior local brain surgery, while 1 patient in phase 2 had received prior radiotherapy for brain metastases. Ten (76.9%) patients (9 SLI, 1 phase 2) had prior systemic anticancer regimens; all except 1 patient had only 1 prior regimen (Table 1). In the SLI, 9 (90.0%) patients had received immunotherapy, primarily nivolumab monotherapy (*n* = 8 [80.0%]); 1 patient received nivolumab and ipilimumab. The median time from the end of the last nivolumab therapy to the start of the study was 1.8 months. In phase 2, 1 patient had previously received other anticancer therapy, and the time from the end of the previous therapy to the start of phase 2 was 23.3 months. In the SLI, 6 (60.0%) patients received a glucocorticoid as a concomitant medication, primarily prednisone (*n* = 5 [50.0%]). In phase 2, 1 (33.3%) patient received both methylprednisolone and prednisolone as concomitant medication (Table 1).

### Patient Disposition

In the SLI, 8 (80.0%) patients discontinued treatment due to radiological disease progression, 1 (10.0%) due to an AE, and 1 (10.0%) due to investigator decision (Supplementary Table 1). In phase 2, all patients (*n* = 3) discontinued treatment due to radiological disease progression (Supplementary Table 1). The main reason for study discontinuation was death (7 [70.0%] patients in the SLI and 3 [100%] patients in phase 2; Supplementary Table 1).

### Determining the RP2D

Nine patients in the SLI who received the high-dose encorafenib regimen were evaluable for DLT assessment and 3 patients (33.3%) experienced DLTs. One patient experienced grade 3 diarrhea and was unable to tolerate ≥40% of the planned dose during Cycle 1, despite the optimal use of antidiarrheal therapy. Two patients were unable to tolerate ≥75% of the planned dose during Cycle 1 (1 each of grade 2 nausea and grade 2 pyrexia; Table 2). Therefore, the high-dose regimen was deemed not tolerable. The standard dose of encorafenib 450 mg QD in combination with binimetinib 45 mg BID was chosen as the RP2D, with intrapatient dose escalation to encorafenib (600 mg QD) with binimetinib (45 mg BID) at Cycle 2 Day 1 for patients who tolerated the standard dose.

**Table 2.** Dose interruptions and reductions

Preferred Term	Safety Lead-In, *n* = 10 *n* (%)	Phase 2, *n* = 3 *n* (%)
No. of patients with any treatment-emergent adverse event leading to encorafenib dose interruption	6 (60.0)	1 (33.3)
Alanine aminotransferase increased	3 (30.0)	1 (33.3)
Aspartate aminotransferase increased	2 (20.0)	1 (33.0)
Abdominal pain	2 (20.0)	0
Diarrhea	1 (10.0)	1 (33.3)
Nausea	2 (20.0)	0
Pyrexia	2 (20.0)	0
Abdominal pain upper	1 (10.0)	0
Acute kidney injury	1 (10.0)	0
Air embolism	0	1 (33.3)
Bronchitis	1 (10.0)	0
Chills	1 (10.0)	0
Decreased appetite	1 (10.0)	0
Dehydration	1 (10.0)	0
Fatigue	1 (10.0)	0
Inappropriate antidiuretic hormone secretion	1 (10.0)	0
Muscular weakness	1 (10.0)	0
Paresthesia	1 (10.0)	0
Peripheral sensory neuropathy	1 (10.0)	0
Rash maculo-papular	1 (10.0)	0
Vomiting	1 (10.0)	0
No. of patients with any treatment-emergent adverse event leading to binimetinib dose interruption	7 (70.0)	1 (33.3)
Alanine aminotransferase increased	3 (30.0)	1 (33.3)
Aspartate aminotransferase increased	2 (20.0)	1 (33.3)
Abdominal pain	2 (20.0)	0
Diarrhea	1 (10.0)	1 (33.3)
Nausea	2 (20.0)	0
Pyrexia	2 (20.0)	0
Abdominal pain upper	1 (10.0)	0
Acute kidney injury	1 (10.0)	0
Air embolism	0	1 (33.3)
Blood creatine phosphokinase increased	1 (10.0)	0
Bronchitis	1 (10.0)	0
Chills	1 (10.0)	0
Decreased appetite	1 (10.0)	0
Dehydration	1 (10.0)	0
Fatigue	1 (10.0)	0
Inappropriate antidiuretic hormone secretion	1 (10.0)	0
Muscular weakness	1 (10.0)	0
Paresthesia	1 (10.0)	0
Peripheral sensory neuropathy	1 (10.0)	0
Rash maculo-papular	1 (10.0)	0
Vomiting	1 (10.0)	0
No. of patients with any treatment-emergent adverse event leading to encorafenib dose reduction, *n* (%)	4 (40.0)	1 (33.3)
Nausea	2 (20.0)	1 (33.3)
Alanine aminotransferase increased	1 (10.0)	1 (33.3)
Aspartate aminotransferase increased	1 (10.0)	1 (33.3)
Diarrhea	1 (10.0)	1 (33.3)
Abdominal pain	1 (10.0)	0
Acute kidney injury	1 (10.0)	0
Decreased appetite	1 (10.0)	0
Fatigue	1 (10.0)	0
Pyrexia	1 (10.0)	0
Rash maculo-papular	1 (10.0)	0
Vomiting	0	1 (33.3)
No. of patients with any treatment-emergent adverse event leading to binimetinib dose reduction, *n* (%)	4 (40.0)	1 (33.3)
Alanine aminotransferase increased	1 (10.0)	1 (33.3)
Aspartate aminotransferase increased	1 (10.0)	1 (33.3)
Nausea	2 (20.0)	0
Abdominal pain	1 (10.0)	0
Acute kidney injury	1 (10.0)	0
Decreased appetite	1 (10.0)	0
Diarrhea	1 (10.0)	0
Fatigue	1 (10.0)	0
Pyrexia	1 (10.0)	0
Rash maculo-papular	1 (10.0)	0

### Safety and Tolerability

Most patients in the overall population (*n* = 12 [92%]) experienced a treatment-related adverse event (TRAE) of any grade, the most frequent (≥30%) being diarrhea (*n* = 7 [54%]), alanine aminotransferase (ALT) increased (*n* = 6 [46%]), fatigue (*n* = 5 [38%]), nausea (*n* = 5 [38%]), and abdominal pain, aspartate aminotransferase (AST) increased, headache, and vomiting (*n* = 4 [31%], each). In the SLI, 4 patients reported grade 3 TRAEs which included diarrhea, AST increased, ALT increased, dehydration, and lymphocyte decreased (Table 3). In phase 2, 3 patients reported grade 3 TRAEs which included diarrhea, nausea, and AST increased. A grade 4 TRAE of ALT increased was reported in 1 patient (Table 3). There were no treatment-related deaths.

**Table 3.** Safety summary: TRAEs experienced by ≥10% of participants in at least 1 cohort for phase 2 by preferred term

	Total, *N* = 13	Safety Lead-In, *n* = 10		Phase 2, *n* = 3	
Preferred Term	All Grades	All Grades	Grades 3–4	All Grades	Grades 3–4
No. of patients with any treatment-related adverse events, *n* (%)	12 (92.3)	9 (90.0)	4 (40.0)	3 (100.0)	1 (33.3)
Diarrhea	7 (53.8)	5 (50.0)	1 (10.0)	2 (66.7)	1 (33.3)
Alanine aminotransferase increased	6 (46.2)	4 (40.0)	1 (10.0)	2 (66.7)	1 (33.3)
Fatigue	5 (38.5)	4 (40.0)	0	1 (33.3)	0
Nausea	5 (38.5)	3 (30.0)	0	2 (66.7)	1 (33.3)
Abdominal pain	4 (30.8)	3 (30.0)	0	1 (33.3)	0
Aspartate aminotransferase increased	4 (30.8)	2 (20.0)	1 (10.0)	2 (66.7)	1 (33.3)
Headache	4 (30.8)	3 (30.0)	0	1 (33.3)	0
Vomiting	4 (30.8)	3 (30.0)	0	1 (33.3)	0

TRAE, treatment-related adverse event.

In the SLI, TEAEs leading to dose interruption of both encorafenib and binimetinib occurred in 6 of 10 patients and those leading to dose reductions of both encorafenib and binimetinib occurred in 4 of 10 patients (Table 2). In phase 2, TEAEs leading to dose interruption and dose reduction of both encorafenib and binimetinib occurred in 1 of 3 patients. An overview of TEAEs leading to dose interruption and dose interruption of encorafenib and of binimetinib is shown in Table 2.

### Efficacy

**Figure 2.**
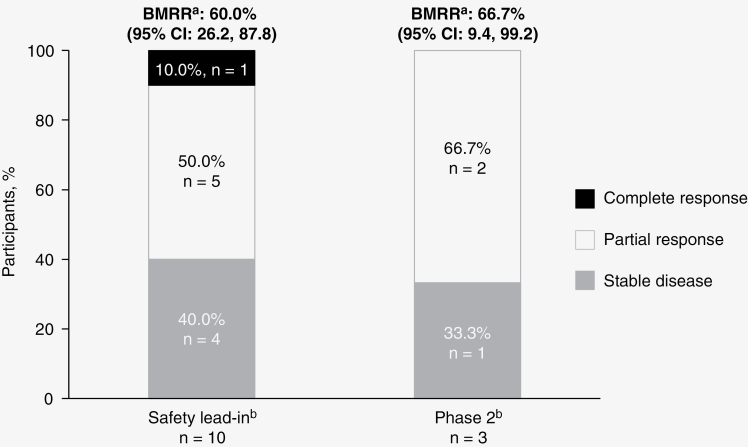
Efficacy results for the safety lead-in and phase 2 parts. Best response after safety lead-in high-dose (encorafenib 300 mg BID + binimetinib 45 mg BID) and phase 2 standard-dose (encorafenib 450 mg QD + binimetinib 45 mg BID) with intrapatient dose escalation (encorafenib 600 mg QD + binimetinib 45 mg BID). Stacked bar graph depicting tumor response in patients with BRAF V600-mutant melanoma with brain metastases. BID, twice daily: BMRR, brain metastasis response rate; QD, once daily. ^a^BMRR is per modified RECIST 1.1. ^b^Graph represents BMRR per modified RECIST 1.1.

In the SLI, the intracranial BMRR was 60.0% (95% CI: 26.2, 87.8), with 1 CR and 5 PRs (Figure 2 and Table 4). In phase 2 Cycle 2, 2 patients were dose escalated and 1 patient remained at the standard dose. One dose-escalated patient and the standard-dose patient achieved PRs, yielding an intracranial confirmed BMRR of 66.7% (95% CI: 9.4, 99.2; Figure 2 and Table 4). The median DOR for brain metastases in the SLI was 3.3 months (95% CI: 2.8, 8.5). In phase 2, 1 responder had a DOR of 6.2 months and the other a DOR of 5.0 months (Figure 2).

**Table 4.** Summary of response

	Safety Lead-In, *n* = 10	Phase 2, *n* = 3
Brain metastasis response rate, *n* (%) [95% CI]	6 (60.0) [26.2, 87.8]	2 (66.7) [9.4, 99.2]
Best overall response, *n* (%)		
Complete response	1 (10.0)	0
Partial response	5 (50.0)	2 (66.7)
Stable response	4 (40.0)	1 (33.3)
Progressive disease	0	0
Not evaluable	0	0
Extracranial response rate, *n* (%) [95% CI]	6 (60.0) [26.2, 87.8]	3 (100.0) [29.2, 100.0]
Best overall response, *n* (%)		
Complete response	0	0
Partial response	6 (60.0)	3 (100.0)
Stable response	1 (10.0)	0
Progressive disease	0	0
Not evaluable	3 (30.0)	0
Global response rate, *n* (%) [95% CI]	5 (50.0) [18.7, 81.3]	3 (100.0) [29.2, 100.0]
Best overall response, *n* (%)		
Complete response	0	0
Partial response	5 (50.0)	3 (100.0)
Stable response	4 (40.0)	0
Progressive disease	0	0
Not evaluable	1 (10.0)	0

The extracranial response rate in the SLI was 60.0% (95% CI: 26.2, 87.8), with 6 patients achieving confirmed PRs. All patients in phase 2 (*n* = 3) also achieved confirmed PRs, for an extracranial response rate of 100% (95% CI: 29.2, 100). Considering best overall brain metastasis and extracranial responses, global response occurred in 5 patients (50.0%) in the SLI and 3 (100%) in phase 2; all were PRs. The median DOR for global response was 2.9 months (95% CI: 2.8, 8.5 months) in the SLI and 5.0 months (95% CI: 3.9, 6.2 months) in phase 2. Due to the low number of patients and early trial termination, overall survival and progression-free survival were not analyzed.

### Pharmacokinetics

The PK parameters from the SLI for encorafenib and LHY746 and binimetinib and AR00426032 are presented in Supplementary Tables 2 and 3, respectively. Following BID administration of encorafenib and binimetinib, geometric mean (geometric coefficient of variation) C_max_ and AUC_tau_ for encorafenib on Day 15 were 1370 (79.3%) and 7490 (52.8%), respectively. Encorafenib exposure after multiple doses was lower at Day 15 than at Day 1, consistent with the CYP3A auto-induction phenomenon observed in historical studies.^32,35^ Binimetinib exposures were consistent with historical studies following single- and repeat-dose administration.^32^ The PK data for the 600 mg QD dose in Phase 2 was not analyzed as there was a lack of sufficient sampling to generate accurate PK parameters.

## Discussion

Recommended therapy for patients with metastatic melanoma with a *BRAF* V600-mutation includes combination regimens targeting BRAF and MEK (eg, dabrafenib plus trametinib, vemurafenib plus cobimetinib, or encorafenib plus binimetinib). The POLARIS study was conducted to further explore regimens of encorafenib in combination with binimetinib in patients with *BRAF* V600-mutant melanoma with asymptomatic brain metastases. While POLARIS was terminated early due to poor accrual for the phase 2 portion, the data collected still provide insights into the safety and antitumor activity of high- and standard-dose encorafenib in combination with binimetinib in this patient population. In POLARIS, the SLI high-dose regimen with encorafenib 300 mg BID when started upfront was not well tolerated, albeit in a largely immunotherapy-experienced population of patients. Thus, the RP2D was determined to be the standard encorafenib dosing (450 mg QD). Intracranial responses were observed in the majority (60%) of patients, with the recognition that patient numbers were small due to early trial termination.

In this study, AEs such as diarrhea, AST increased, fatigue, and nausea were common in both the SLI and phase 2. In phase 2 there were no new or unexpected toxicities for the standard or intrapatient escalation dose of encorafenib in combination with binimetinib.

In the SLI population, 1 patient achieved a confirmed CR, and the intracranial response rate was (67%). While DORs were short, they were consistent with the COMBI-MB trial experience for dabrafenib (BRAF inhibitor) plus trametinib (MEK1/2 inhibitor),^26^ although direct comparisons are difficult due to the small number of patients enrolled in the POLARIS trial and cross-trial differences. It is notable that the POLARIS trial also enrolled patients with asymptomatic brain metastases, similar to previous studies, although we note that cross-trial comparisons should not be made.^26^ Additionally, in a small retrospective case series, combination therapy with encorafenib plus binimetinib elicited an intracranial objective response rate of 33%; however, this report included patients previously treated with BRAF/MEK inhibitors.^36^ The blood–brain barrier may be compromised in patients with brain metastases, which may explain the decreased intracranial drug levels noted and the decreased efficacy, including in DOR. For example, dabrafenib and trametinib are efflux transporter substrates thought to have limited brain penetration based on nonclinical data; however, the COMBI-MB trial found that the standard dosing of the combination had clinical intracranial activity, although with short DORs.^26,37,38^

A dose of 300 mg BID was investigated since the 600 mg QD dose was tolerated well by most patients treated with this dose in the phase 1 trial. However, this dose was not selected as the maximum tolerated dose due to rare renal toxicity.^32^ The high-dose steady-state daily exposure of encorafenib 300 mg BID (in combination with binimetinib) was higher compared with historical studies with 300 mg QD dosing.^3,35^ Furthermore, compared to standard dosing, daily exposure for encorafenib 300 mg BID was similar to 450 mg QD.^3,32^ These data indicate that increasing encorafenib dosing frequency is unlikely to account for the toxicity seen with the SLI regimen. Similar to a previous report in a retrospective study of (standard dose) BRAF inhibition combined with MEK inhibition in patients with melanoma who had already received immunotherapy,^39^ the high rate of AEs observed in this study of encorafenib in combination with binimetinib for patients who had previously received anti-PD-1–based immunotherapy may have been partially due to the short time between their last immunotherapy dose and the beginning of their new treatment.

In conclusion, these are the first data for encorafenib in combination with binimetinib evaluating a high-dose regimen in patients with *BRAF* V600-mutant melanoma with brain metastasis. In this study several dosage levels were examined, with signs of intracranial activity. Results from the POLARIS trial are in alignment with the current understanding of treatment refractiveness in this patient population. However, DOR was limited, and data interpretation was restricted due to the small number of patients in each part of the study. Unexpectedly, treatment with higher-dose encorafenib (300 mg BID) did not result in increased drug exposure, likely due to increased drug metabolism. The tolerability of the standard encorafenib dose in combination with binimetinib was consistent with previous experience, with similar PK and no new safety signals.^32^ These results highlight an important unmet need to develop systemic therapies, especially after progression on immunotherapy, to further improve outcomes in patients with melanoma brain metastases.

## Supplementary Material

vdae033_suppl_Supplementary_Data

vdae033_suppl_Supplementary_Tables_S1

vdae033_suppl_Supplementary_Tables_S2

vdae033_suppl_Supplementary_Tables_S3

## Data Availability

Upon request, and subject to review, Pfizer will provide the data that support the findings of this study. Subject to certain criteria, conditions, and exceptions, Pfizer may also provide access to the related individual de-identified patient data. See https://www.pfizer.com/science/clinical-trials/trial-data-and-results for more information.
