# Supplementary material for: POLARIS: A phase 2 trial of encorafenib plus binimetinib evaluating high-dose and standard-dose regimens in patients with BRAF V600-mutant melanoma with brain metastasis
Source: Neurooncol Adv. 2024 Mar 18;6(1):vdae033. doi: 10.1093/noajnl/vdae033 (PMC11079948; doi:10.1093/noajnl/vdae033)
Supplement: vdae033_suppl_Supplementary_Data [file vdae033_suppl_Supplementary_Data.docx]

**Supplementary data**

*Supplementary methods*

Study design and patients

Patients may have received prior checkpoint immunotherapy (e.g., CTLA-4 or PD-1 antibodies) but no previous BRAF/MEK inhibitors. All prohibited concomitant medication washout periods must have been completed before a screening period started or 28 days before first dose. After a screening period, treatment was administered in 28-day cycles and continued until disease progression, unacceptable toxicity, withdrawal of consent, the start of subsequent anticancer therapy, or death. Once a patient discontinued study treatment, the treatment period ended and the patient entered the follow-up period.

This high-dose regimen would be considered safe if the dose-limiting toxicity (DLT) rate was <33%, in which case, patients would have been randomized to receive high- or standard-dose treatment. Otherwise, standard-dose encorafenib 450 mg once daily plus binimetinib 45 mg twice daily would be selected as the recommended phase 2 dose. DLT-evaluable patients for the safety lead-in (SLI) must have experienced either a DLT or received ≥75% of the planned cumulative dose of both study drugs during SLI Cycle 1. Patients who remained on treatment at the end of the study and who, in the investigator’s opinion, derived benefit from continued treatment with encorafenib in combination with binimetinib were allowed to continue treatment in accordance with local regulations.

Key inclusion criteria

Patients must have had ≥1 parenchymal brain lesions ≥0.5 cm and ≤ 4 cm, defined as a magnetic resonance imaging contrast-enhancing lesion that may be accurately measured in ≥1 dimension. In the SLI and phase 2, patients may have received prior local therapy for brain metastases, including but not restricted to brain surgery, whole brain radiotherapy, stereotactic radiotherapy or stereotactic radiosurgery (e.g., gamma knife, linear-accelerated-based radiosurgery, charged particles, and CyberKnife). In the SLI, BRAF or MEK inhibitor(s) received ≥12 months prior to enrollment in the adjuvant setting were allowed; in phase 2, ≥6 months prior to enrollment in the adjuvant setting was required.

Key exclusion criteria

Patients on prior immunotherapy were permitted and may have received BRAF or MEK inhibitors in the adjuvant setting provided that the treatment ended ≥6 months prior to enrollment. Patients treated in the adjuvant setting with BRAF or MEK inhibitors <6 months prior to enrollment. Prior corticosteroid treatment for brain metastases was allowed if the patient was on a stable or decreasing dose (≤4 mg total daily dose of dexamethasone or equivalent) for ≥2 weeks before first study treatment.

Safety analysis

Adverse events (AEs) were coded using the MedDRA®. Incidence of AEs by maximum severity, serious AEs, AEs assessed as related to study drug, and AEs resulting in discontinuation of study drug were recorded.

Pharmacokinetic analysis

Blood samples (≈4 mL each) for plasma pharmacokinetic (PK) analysis of encorafenib and its metabolite (LHY746), as well as binimetinib and its metabolite (AR00426032) were collected from all patients enrolled in the SLI and phase 2 parts. In the SLI, samples were collected on Cycle 1 Day 1 (C1D1) at 0.5 and 1.5 hours (±5 minutes), 3.0 hours (±10 minutes), and 6 hours (±20 minutes) postdose. Samples were collected on Cycle 1 Day 15 (C1D15) at the same timepoints as C1D1 with the addition of predose (within 30 minutes of dose).

If the high-dose regimen was tested in phase 2, subsequent patient blood samples were to be collected pre- and postdose on C1D15, and predose on Cycle 2 Day 1 (C2D1) and Cycle 3 Day 1 (C3D1); if only the standard-dose regimen was tested, subsequent samples were to be collected predose and postdose on C2D1 and C3D1. Plasma concentrations were determined using a quantitaive liquid chromatography with tandem mass spectrometry (LC-MS-MS, Triple Quad 6500, AB SCIEX) and plasma concentration-time profiles were generated.

PK parameters were determined for all PK-evaluable patients using noncompartmental method(s). The PK parameters reported, when feasible and appropriate, included were maximum (C_max_), measured concentration at the end of a dosing interval (C_trough_), time to reach C_max_ (T_max_), area under the plasma concentration-time curve over the dosing interval (AUC_tau_) and area under the plasma concentration-time curve from zero to 6 hours (AUC_0-6_), which were all observed directly from data. Area under the plasma concentration-time curve from zero to the last measurable time point (AUC_last_) was calculated using the linear up/log down interpolation method and accumulation ratio and was calculated as C1D15 AUC_0-6_/C1D1 AUC_0-6_ (R_AUC_). Dose-normalized parameters were calculated as appropriate for C_max_, C_trough_, and AUC_last_. Dose-normalized AUC_last_ values as well as R_AUC_ values for the standard- and high-dose treatment arms were compared if data permitted.

Statistical analyses

Secondary efficacy endpoints included: 1) the extracranial response rate, defined as the proportion of patients with a best overall extracranial response of confirmed CR or confirmed PR according to RECIST 1.1 per investigator assessment; 2) the global response rate, defined as the proportion of patients with a best overall response of confirmed CR or confirmed PR for brain metastasis and extracranial lesions; and 3) the DOR (brain metastasis, extracranial, or global) defined as the time from the first documented CR or PR that were subsequently confirmed to disease progression or death due to any cause, whichever occurs first. For DOR, a patient would be censored at the date of last adequate tumor assessment in the absence of disease progression or death at the time of the analysis cutoff or at the start of any new anticancer therapy.

The corresponding exact Clopper-Pearson 2-sided 95% CIs of the BMRR were calculated. The primary analysis took place after all patients in the efficacy population had ≥2 postbaseline tumor assessments and after all patients with an initial response were followed up for ≥6 months.
