# Supplementary material for: POLARIS: A phase 2 trial of encorafenib plus binimetinib evaluating high-dose and standard-dose regimens in patients with BRAF V600-mutant melanoma with brain metastasis
Source: Neurooncol Adv. 2024 Mar 18;6(1):vdae033. doi: 10.1093/noajnl/vdae033 (PMC11079948; doi:10.1093/noajnl/vdae033)
Supplement: vdae033_suppl_Supplementary_Tables_S1 [file vdae033_suppl_Supplementary_Tables_S1.docx]

**Supplementary Table S1. Patient disposition (safety set)**

| **Disposition** | **All Patients**  **N=13**  **N (%)** | **Safety Lead-in**  **n=10**  **n (%)** | **Phase 2**  **n=3**  **n (%)** |
| --- | --- | --- | --- |
| Patients treated | 13 (100) | 10 (100) | 3 (100) |
| Treatment discontinued | 13 (100) | 10 (100) | 3 (100) |
| Primary reason for treatment discontinuation |  |  |  |
| Adverse event | 1 (7.7) | 1 (10.0) | 0 |
| Disease progression (radiological) | 11 (84.6) | 8 (80.0) | 3 (100) |
| Investigator decision | 1 (7.7) | 1 (10.0) | 0 |
| Study evaluation after treatment discontinuation |  |  |  |
| Patients who discontinued the study | 13 (100) | 10 (100) | 3 (100) |
| Primary reason for study discontinuation |  |  |  |
| Death | 10 (76.9) | 7 (70.0) | 3 (100) |
| Study termination by sponsor | 2 (15.4) | 2 (20.0) | 0 |
| Other | 1 (7.7) | 1 (10.0) | 0 |
