# Supplementary material for: POLARIS: A phase 2 trial of encorafenib plus binimetinib evaluating high-dose and standard-dose regimens in patients with BRAF V600-mutant melanoma with brain metastasis
Source: Neurooncol Adv. 2024 Mar 18;6(1):vdae033. doi: 10.1093/noajnl/vdae033 (PMC11079948; doi:10.1093/noajnl/vdae033)
Supplement: vdae033_suppl_Supplementary_Tables_S2 [file vdae033_suppl_Supplementary_Tables_S2.docx]

**Supplementary Table S2. Pharmacokinetic parameters of encorafenib and metabolite in the safety lead-in^a^**

|  | **Encorafenib^b^** | | | | **LHY746^b^** | | | |
| --- | --- | --- | --- | --- | --- | --- | --- | --- |
| **Parameter (unit)** | **n^c^** | **C1D1**  **n=9** | **n^c^** | **C1D15**  **n=8** | **n^c^** | **C1D1**  **n=9** | **n^c^** | **C1D15**  **n=8** |
| AUC_0-6_, (%CV)^d^, ng*h/mL | 7 | 9530 (50.6) | 6 | 3930 (52.8) | 7 | 1230 (60.1) | 6 | 8160 (67.7) |
| AUC_last_ (%CV), ng*h/mL | 8 | 9190 (47.8) | 6 | 7490 (52.8) | 8 | 1180 (56.9) | 6 | 29600 (73.0) |
| AUC_tau_ (%CV), ng*h/mL | NA | NA | 6 | 7490 (52.8) | NA | NA | 6 | 29600 (73.0) |
| C_max_ (%CV), ng/mL | 8 | 3210 (47.7) | 6 | 1370 (79.3) | 8 | 340 (47.2) | 6 | 1720 (65.3) |
| C_trough_ (%CV), ng/mL | NA | NA | 6 | 332 (61.0) | NA | NA | 6 | 1520 (59.0) |
| T_max_ (min−max), h | 8 | 1.53 (1.47−3.00) | 6 | 1.55 (0.43−3.00) | 8 | 4.33 (1.47−6.00) | 6 | 3.00 (2.92−5.73) |
| R_AUC_^e^ (%CV) | NA | NA | 6 | 0.468 (46.0) | NA | NA | 6 | 7.25 (46.7) |
| R_Cmax_^e^ (%CV) | NA | NA | 6 | 0.490 (71.8) | NA | NA | 6 | 5.78 (41.7) |

AUC_0-6_, area under the concentration curve from 0 to 6 hours; AUC_last_, area under the concentration curve from dosing to the last measurable concentration; AUC_tau_, area under the plasma concentration-time curve over the dosing interval; BID, twice daily; C1D1, Cycle 1 Day 1; C1D15, Cycle 1 Day 15; C_max_, maximum serum concentration; C_trough_, trough plasma concentration; CV, coefficient of variation; NA, not applicable; PK, pharmacokinetics; R, accumulation ratio; SLI, safety lead-in; T_max_, time to maximum plasma concentration. ^a^ All 13 patients were included in the PK set, which includes all patients who receive ≥1 dose of any study drug and have ≥1 PK blood collection after the first dose of study drug with associated bioanalytical results. However, the 3 patients in phase 2 were split into cohort 1 (n=1, prior therapy) and cohort 2 (n=2, no prior therapy), and sampling was not sufficient to support noncompartmental analysis in any patient. As a result, only PK data from the 10 participants in the SLI arm are presented. ^b^ All patients were assigned to the high-dose treatment of encorafenib 300 mg BID plus binimetinib 45 mg BID. ^c^ Number of patients with nonmissing values. ^d^ %CV is geometric mean CV. ^e^ Accumulation ratios were calculated as: C1D15 AUC_0-6_ or C_max_ divided by C1D1 AUC_0-6_ or C_max._
