# Supplementary material for: POLARIS: A phase 2 trial of encorafenib plus binimetinib evaluating high-dose and standard-dose regimens in patients with BRAF V600-mutant melanoma with brain metastasis
Source: Neurooncol Adv. 2024 Mar 18;6(1):vdae033. doi: 10.1093/noajnl/vdae033 (PMC11079948; doi:10.1093/noajnl/vdae033)
Supplement: vdae033_suppl_Supplementary_Tables_S3 [file vdae033_suppl_Supplementary_Tables_S3.docx]

**Supplementary Table S3. Pharmacokinetic parameters for binimetinib and metabolite in the safety lead-in^a^**

|  | **Binimetinib^b^** | | | | **AR00426032^b^** | | | |
| --- | --- | --- | --- | --- | --- | --- | --- | --- |
| **Parameter (unit)** | **n^c^** | **C1D1**  **n=9** | **n^c^** | **C1D15**  **n=8** | **n^c^** | **C1D1**  **n=9** | **n^c^** | **C1D15**  **n=8** |
| AUC_0-6_, (%CV)^d^, ng*h/mL | 7 | 1410 (85.5) | 6 | 1050 (39.7) | 7 | 159 (65.9) | 6 | 53.9 (48.6) |
| AUC_last_ (%CV), ng*h/mL | 8 | 1350 (79.1) | 6 | 1440 (43.9) | 8 | 156 (60.6) | 6 | 77.9 (49.7) |
| AUC_tau_ (%CV), ng*h/mL | NA | NA | 6 | 1440 (43.9) | NA | NA | 6 | 77.9 (49.7) |
| C_max_ (%CV), ng/mL | 8 | 506 (85.5) | 6 | 359 (40.5) | 8 | 53.9 (77.8) | 6 | 16.9 (54.0) |
| C_trough_ (%CV), ng/mL | NA | NA | 6 | 79.9 (56.6) | NA | NA | 6 | 4.71 (62.8) |
| T_max_ (min−max), h | 8 | 1.50 (1.45−6.08) | 6 | 1.55 (0.43−2.98) | 8 | 1.53 (1.47−6.08) | 6 | 1.58 (0.43−3.07) |
| R_AUC_^e^ (%CV) | NA | NA | 6 | 0.877 (60.4) | NA | NA | 6 | 0.359 (93.0) |
| R_Cmax_^e^ (%CV) | NA | NA | 6 | 0.818 (95.9) | NA | NA | 6 | 0.347 (130.9) |

AUC_0-6_, area under the concentration curve from 0 to 6 hours; AUC_last_, area under the concentration curve from dosing to the last measurable concentration; AUC_tau_, area under the plasma concentration-time curve over the dosing interval; BID, twice daily; C1D1, Cycle 1 Day 1; C1D15, Cycle 1 Day 15; C_max_, maximum serum concentration; C_trough_, trough plasma concentration; CV, coefficient of variation; NA, not applicable; PK, pharmacokinetics; R, accumulation ratio; SLI, safety lead-in; T_max_, time to maximum plasma concentration. ^a^ All 13 patients were included in the PK set, which includes all patients who receive ≥1 dose of any study drug and have ≥1 PK blood collection after the first dose of study drug with associated bioanalytical results. However, the 3 patients in phase 2 were split into cohort 1 (n=1, prior therapy) and cohort 2 (n=2, no prior therapy), and sampling was not sufficient to support noncompartmental analysis in any patient. As a result, only PK data from the 10 participants in the SLI arm are presented. ^b^ All patients were assigned to the high-dose treatment of encorafenib 300 mg BID plus binimetinib 45 mg BID. ^c^ Number of patients with nonmissing values. ^d^ %CV is geometric mean CV. ^e^ Accumulation ratios were calculated as: C1D15 AUC_0-6_ or C_max_ divided by C1D1 AUC_0-6_ or C_max_
